# Generalised versus Regional Odontodysplasia: Diagnosis, Transitional Management, and Long-Term Followup—A Report of 2 Cases

**DOI:** 10.1155/2013/519704

**Published:** 2013-02-14

**Authors:** Mostafa Ibrahim Mostafa, Nehad Samir Taha, Mennat Allah Ismail Mehrez

**Affiliations:** ^1^Department of Oro-Dental Genetics, National Research Centre, 33 Tahrir Street, Dokki, Cairo 12311, Egypt; ^2^Oral Histopathology Department, Misr International University (MIU), Cairo, Egypt

## Abstract

*Background*. Odontodysplasia is an uncommon condition. It can be localised or generalised, isolated or part of a syndrome. *Case Report*. We first report the case of an 8.5-year-old boy who presented with abnormally shaped teeth and multiple intraoral abscesses. Findings of clinical and radiographic examinations were consistent with those of generalised odontodysplasia. A 2-step, conservative treatment plan was executed with a 10-year followup. Step 1 was root canal treatment of nonvital teeth. Step 2 was crowning without preparation of new vital, erupted, and malformed teeth. The second case is that of a 12-year-old girl who presented with lack of teeth in the upper-left quadrant. The impacted teeth were exposed surgically, and fixed restoration was performed for temporary aesthetic improvement. *Conclusion*. Instead of tooth extraction, an approach previously used in similar cases, conservative management is feasible and offers better prosthetic treatment options for the future.

## 1. Introduction

Odontodysplasia (OD) is an uncommon condition that affects the enamel and dentine of both primary and permanent dentitions. The cementum shows affection, but at a lower grade. The teeth are misshaped, pitted, and yellowish to brownish in colour, with excessive wear and underlying periapical abscesses. On radiographs, the enamel and dentine lack contrast, rendering the tooth a ghostlike appearance [1, 2].

This condition commonly presents as regional OD (ROD), wherein few teeth may be involved. Occasionally, an entire quadrant or more than 1 quadrant may be involved, but generalised involvement is extremely rare [3]. ROD may present as an isolated condition or as part of a syndrome such as oculodentodigital syndrome [OMIM:#164200] or epidermal nevus/Schimmelpenning-Feuerstein-Mims syndrome [OMIM: %163200] [4, 5].

The precise aetiology of OD is still unknown. Genetic predisposition has been proposed, but environmental causation has received greater support [1]. Previously employed treatment plans differed according to the degree of teeth involvement and the patient's age [6–8]. The treatment protocol advocated, provided that the patient is of proper age, is implant placement with a final fixed restoration [9]. 

In this paper, we present 2 cases, one of generalised OD and another with ROD, and propose a protocol for transitional management and long-term followup (10 and 5 years, resp.) of both types of cases.

## 2. Case 1: Generalised Odontodysplasia

**Figure 1:**
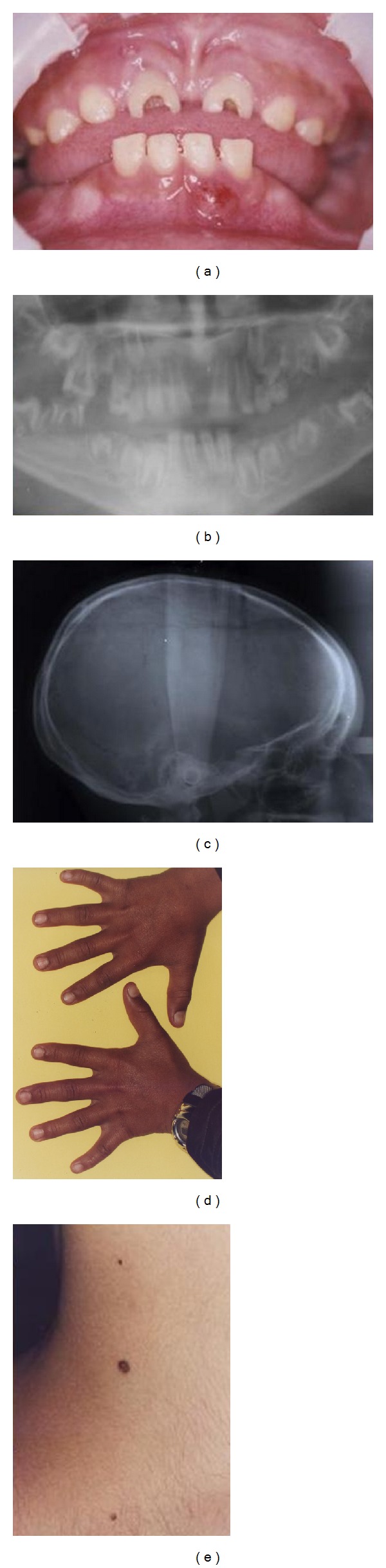
(a) Case 1. Oral condition of GOD at time of presentation. (b) Panoramic view showing ghost-shaped teeth, enlarged pulp chambers and root canals, and short roots and open apices. (c) Lateral cephalometric view depicting dolichocephaly. (d) Bilateral clinodactyly of the little fingers. (e) Multiple nevi on the neck.

An 8.5-year-old boy presented complaining of abnormally shaped teeth and multiple intraoral swellings. Oral examination revealed severe wear of the upper centrals, upper first molars, and the lower-right first molar, with no carious lesions. The lower-right first molar was mutilated beyond repair and was indicated for extraction. Abscesses and fistulas were related to upper-right and lower-left incisors. Microdontia was also evident (Figure 1(a)).

The patient reported that he had had delayed eruption and premature loss of the primary dentition and that the lower-left first molar had been extracted. Vitality testing revealed that all incisors and the upper-right first and second molars were nonvital.

A panoramic radiograph showed ghost-shaped teeth. The roots had enlarged pulp chambers and root canals, short roots, and open apices (Figure 1(b)).

The patient was dolichocephalic (Figure 1(c)). He had clinodactyly and multiple nevi on both his neck and back (Figures 1(d) and 1(e)). Positive consanguinity was present with negative family history. Maternal pregnancy and delivery history were uneventful, and there was no evidence of any of the suggested causative factors.

**Figure 2:**
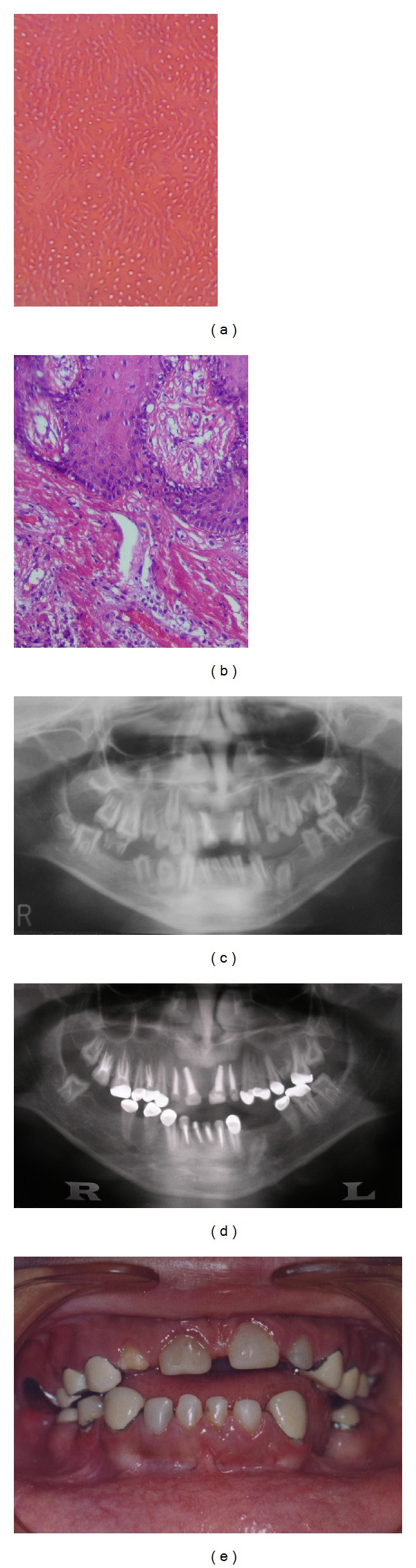
(a) Histological examination showing dentin having torturous dentinal tubules and globular masses of dentine interrupting them (original magnification 20x). (b) Gingival biopsy examination showing odontogenic tissue in the epithelium and intramesenchymal calcifications (original magnification 20x). (c) Panoramic view showing apexification trial. (d) RCT without crowning was done for incisors and upper-right second molar. RCT with crowning was performed for upper-right second premolar and first molar and upper-left first premolar. Crowning without RCT was done for the vital upper-left premolars, left second molar, lower left second premolar, lower left second molar, lower canines, right first premolar, and upper-right first molar. (e) Final oral condition at 18 years of age.

Histopathologic examination of the extracted upper-right first molar revealed that the dentine had tortuous dentinal tubules and globular masses of dentine interrupting them (Figure 2(a)). Gingival biopsy examination, on the other hand, showed odontogenic tissue in the epithelium and intramesenchymal calcifications (Figure 2(b)).

The patient had normal levels of alkaline phosphatase and serum calcium and phosphorus. Based on the clinical and radiographic findings, generalised OD was diagnosed.

*Management*. Apexification of the upper centrals was first attempted over a period of 2 years but failed (Figure 2(c)). We formulated a conservative treatment plan to be carried out at the age of 12 years and over a period of 2.5 years. Step 1 was root canal treatment (RCT) of nonvital teeth, including those with open apices and abscesses underneath. Step 2 was crowning without preparation of the new vital, erupted, and malformed teeth, to protect them from the sequelae of poor development. Metal crowns fused to porcelain were used to meet aesthetic requirements, with gingival or subgingival metallic margins to minimise overhang. 

We lost contact with the patient over the following year and a half. During this period, the lower-right second molar was extracted because of severe mutilation and abscess formation. This information was reported by the child's parents.

At the 10-year followup, the 7-year-old RCT was successful. Vitality of crowned teeth was preserved, and these teeth showed apex formation. The lower-left first premolar was still impacted. Radiolucency of the upper-right incisors and the lower-right lateral incisor persisted, although the radiolucent area was markedly smaller in the lower-right lateral incisor (Figures 2(d) and 2(e)).

## 3. Case 2: Regional Odontodysplasia 

**Figure 3:**
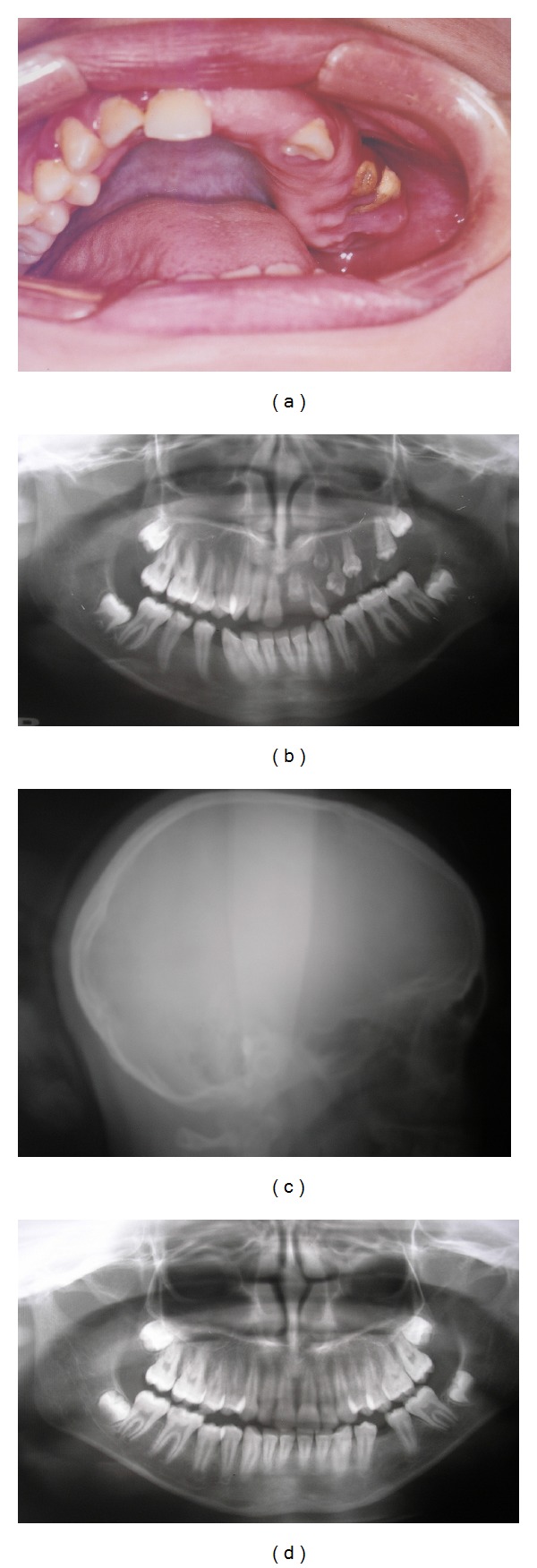
(a) Case 2. Oral condition of ROD of the upper-left quadrant at time of presentation. (b) Panoramic view of Case 2 showing multiple impacted teeth in the upper left quadrant and a missing lower right first premolar. (c) Lateral cephalometric view depicting dolichocephaly. (d) Panoramic view of Case 2 twin brother showing a missing lower left first premolar.

A 12-year-old girl presented to our clinic with lack of teeth in the upper-left quadrant. Examination revealed a canine and a root of a deciduous tooth. At the affected area, the gingiva was enlarged, fibrous, and tense (Figure 3(a)). The lower-right first molar was congenitally absent. Dolichocephaly was noted during the extraoral examination (Figure 3(c)), and there was no evidence of multiple nevi on the skin.

The maternal pregnancy and delivery history were unremarkable. There were no similar cases among the family, and there was no history of any of the suggested causative factors. Patient had osteopenia according to the DEXA scan. A panoramic radiograph revealed multiple impacted teeth in the upper-left quadrant and a missing lower-right first molar (Figure 3(b)). The patient had a twin brother whose teeth had erupted normally, except for the lower-first molar on the left side, which was congenitally absent (Figure 3(d)).

**Figure 4:**
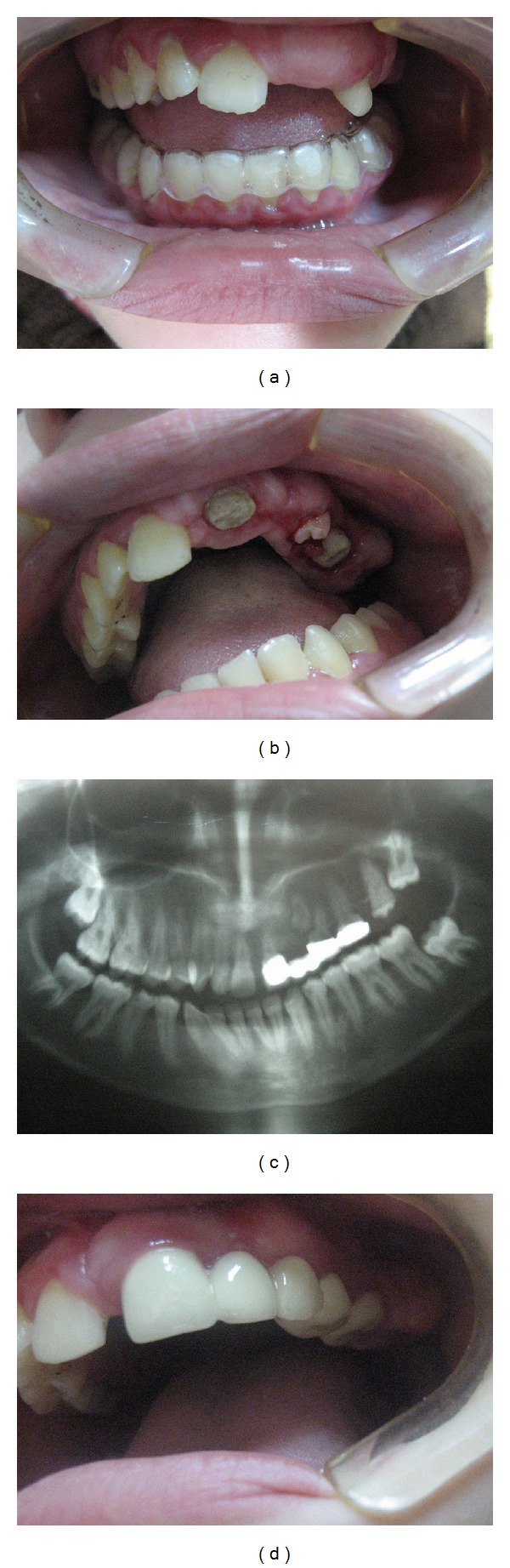
(a) Vacuum formed tray for the lower jaw to prevent overeruption of lower teeth. (b) Surgical exposure of the impacted teeth. (c) Panoramic view for the case after surgical exposure and placement of the porcelain fused to metal bridge. (d) Final oral condition at 17 years of age.

*Management*. The treatment plan was implant placement after completion of the growth period. Prevention of opposing overeruption was necessary, and a vacuum-formed tray was prepared for the lower jaw (Figure 4(a)). 

After 2.5 years, a periapical abscess had developed at the canine, which had become loose. We extracted the canine but were in turn faced by another problem, the increased aesthetic demand of the now 14.5-year-old girl. A change in treatment strategy was mandatory. Surgical exposure of the impacted teeth was performed, followed by porcelain-fused-to-metal fixed restoration as a temporary aesthetic solution (Figure 4(b)).

Three years later, the gingiva at the restoration (as in Case 1) showed signs of inflammation, and a panoramic radiograph showed asymptomatic periapical radiolucency in the upper-left first premolar (Figures 4(c) and 4(d)). The patient was satisfied with the functional and aesthetic results.

## 4. Discussion 

Early diagnosis of OD is critical. The diagnosis is based on clinical and radiographic examination, but extraoral features should not be neglected. Dentinogenesis imperfecta type III bears close resemblance to OD but can be excluded in the absence of opalescent dentine, bell-shaped crowns, and negative family history. Delayed eruption or failure of eruption and gingival enlargement occur only in OD [10].

Generalised OD in Case 1 showed some extraoral features of both oculodentodigital and epidermal nevus syndromes and could have been a mild variant of either syndrome. Dolichocephaly was present in both cases, while clinodactyly was only present in Case 1 (generalised OD). Dolichocephaly and clinodactyly could be associated with OD. Fanibunda and Soames (1996) reported a case of ROD associated with dolichocephaly and clinodactyly [11].

Several hypotheses have been proposed to explain the cause of OD. We agree with the hypothesis of local irritating factors during teeth development because the twin brother of the patient in Case 2 had only the missing premolar and was free from OD. 

Our treatment plan was based on the theory of Gardner and Sapp (1973), who reported that the cause of pulpal necrosis and subsequent periapical abscess is bacterial invasion of the pulp through defective clefts in the enamel and dentine [12, 13]. This explains the completion of root formation and the lack of periapical pathosis in the newly erupted teeth after crowning in Case 1 (Figure 2(d)).

Since the teeth in these cases were not bell shaped, it was possible to crown them without preparation and with only a few fitting adjustments at the time of insertion. The upper and lower incisors were not crowned because early failure was expected.

Regarding ROD in Case 2, the patient did not wear the vacuum tray, but the gingival enlargement acted as a stopper and achieved the purpose of the vacuum tray.

## 5. Conclusion

Maintenance of oral hygiene and periodic checkups are necessary in similar cases. In the authors' opinion, conservative treatment of these patients is preferable to extraction because it preserves optimum oral functionality and aesthetics until the end of the growth period. At that time, implants can be placed safely. Conservative treatment offers better prosthetic options for the future.
